# Improved results of LINE-1 methylation analysis in formalin-fixed, paraffin-embedded tissues with the application of a heating step during the DNA extraction process

**DOI:** 10.1186/s13148-016-0308-0

**Published:** 2017-01-13

**Authors:** Xianyu Wen, Seorin Jeong, Younghoon Kim, Jeong Mo Bae, Nam Yun Cho, Jung Ho Kim, Gyeong Hoon Kang

**Affiliations:** 1 Department of Pathology, Seoul National University College of Medicine, 28 Yongon-dong, Chongno-gu, Seoul, 110-744, South Korea; 2 Laboratory of Epigenetics, Seoul National University College of Medicine, Seoul, South Korea; 3 Department of Pathology, SMG-SNU Boramae Medical Centre, Seoul, South Korea

**Keywords:** Archival tissue, CpG island methylator phenotype, DNA methylation, Formalin, Heat treatment, LINE-1

## Abstract

**Background:**

Formalin-fixed, paraffin-embedded (FFPE) tissues are important resources for profiling DNA methylation changes and for studying a variety of diseases. However, formalin fixation introduces inter-strand crosslinking, which might cause incomplete bisulfite conversion of unmethylated cytosines, which might lead to falsely elevated measurements of methylation levels in pyrosequencing assays. Long interspersed nucleotide element-1 (LINE-1) is a major constituent of repetitive transposable DNA elements, and its methylation is referred to correlates with global DNA methylation. To identify whether formalin fixation might impact the measured values of methylation in LINE-1 repetitive elements and whether prolonged heat-induced denaturation of DNA might reduce the artificial increases in measured values caused by formalin fixation, we analyzed paired fresh-frozen (FF) and FFPE xenograft tissue samples for their methylation levels in LINE-1 using a pyrosequencing assay. To further confirm the effect of a heating step in the measurement of LINE-1 or single gene methylation levels, we analyzed FFPE tissue samples of gastric cancer and colorectal cancer for their methylation status in LINE-1 and eight single genes, respectively.

**Results:**

Formalin fixation led to an increase in the measured values of LINE-1 methylation regardless of the duration of fixation. Prolonged heating of the DNA at 95 °C for 30 min before bisulfite conversion was found (1) to decrease the discrepancy in the measured values between the paired FF and FFPE tissue samples, (2) to decrease the standard deviation of the measured value of LINE-1 methylation levels in FFPE tissue samples of gastric cancer, and (3) to improve the performance in the measurement of single gene methylation levels in FFPE tissue samples of colorectal cancer.

**Conclusions:**

Formalin fixation leads to artificial increases in the measured values of LINE-1 methylation, and the application of prolonged heating of DNA samples decreases the discrepancy in the measured values of LINE-1 methylation between paired FF and FFPE tissue samples. The application of prolonged heating of DNA samples improves bisulfite conversion-based measurement of LINE-1 or single gene methylation levels in FFPE tissue samples.

**Electronic supplementary material:**

The online version of this article (doi:10.1186/s13148-016-0308-0) contains supplementary material, which is available to authorized users.

## Background

LINE-1 is repeated half a million times in the human genome, interspersed throughout, and comprises approximately 17% of the human genome [1]. It has a high density of CpG dinucleotides in its 5′ untranslated region, and these CpG sites are usually heavily methylated in normal cells. Because of both the extremely high frequency of LINE-1 and the heavy methylation in its 5′ CpG sites, the level of LINE-1 methylation has been thought to be closely associated with genomic DNA methylation levels. In 2005, Weisenberger et al. demonstrated a strong relationship between the levels of LINE-1 methylation and genomic DNA methylation [2]. In that study, the LINE-1 methylation level was assessed using the MethyLight assay, a probe-based real-time PCR assay for the detection of methylation. More recently, however, instead of using the MethyLight-based LINE-1 methylation assay, a pyrosequencing-based LINE-1 methylation assay has become widely used because the pyrosequencing-based assay has been validated for its precision and reliability in formalin-fixed, paraffin-embedded (FFPE) tissue samples by Irahara et al. [3].

LINE-1 hypomethylation in tumors has been demonstrated in virtually all tissue types of human cancer except for thyroid cancer and renal cell carcinoma [4–11]. However, prior to the report of Tournier et al. in 2012, there had been no study which compared the results of the PCR-based LINE-1 methylation assay between paired fresh-frozen (FF) and FFPE tissue samples [12]. Tournier and colleagues analyzed paired FF and FFPE tissue samples for their LINE-1 methylation levels using a pyrosequencing assay to identify whether formalin fixation induced deviations in the measured value of methylation levels in individual genes or in LINE-1. Tournier et al. found that a significant discrepancy existed in the measured LINE-1 methylation levels between paired FF and FFPE tissue samples. This discrepancy raised doubts regarding the utility of the pyrosequencing LINE-1 methylation assay in FFPE tissue samples.

In formalin-fixed tissue samples, formaldehyde induces several types of DNA damage on either double strand or single strand, including formaldehyde-induced crosslinks, DNA fragmentation, abasic sites, and deamination of cytosine bases [13]. Of various formaldehyde-induced crosslinks, inter-strand DNA crosslinks and protein-DNA crosslinks are thought to affect the efficacy of bisulfite modification which is an essential step for genomic DNA methylation analysis. Because the reaction of bisulfite with cytosine residues is highly single strand-specific and cannot occur on double-stranded DNA [14], inter-strand DNA crosslinks are thought to cause some resistance against heat and alkaline denaturation. Incomplete denaturation of DNA leads to incomplete bisulfite conversion, which might cause the discrepancy in the measured values of LINE-1 methylation between paired FF and FFPE tissue samples. Because formaldehyde-induced crosslinks are known to be reversible by heat treatment [15, 16], the application of prolonged heat treatment during DNA preparation process might increase the performance of bisulfite modification in DNA samples obtained from FFPE tissues [12]. The present study aimed to identify whether formalin fixation is related to the increased values of LINE-1 methylation detected in FFPE tissues and whether the addition of a heating step during the DNA extraction process helps to decrease the discrepancy in the measured values of LINE-1 methylation between paired FF and FFPE tissue samples. From five cancer cell lines, we generated 10 xenograft samples that were analyzed for the effects of formalin fixation and for heating in the pyrosequencing-based assay of LINE-1 methylation. Finally, we attempted to identify the effect of heating in the methylation analysis of LINE-1 or of single genes in human FFPE tissue samples.

## Results

**Fig. 1:**
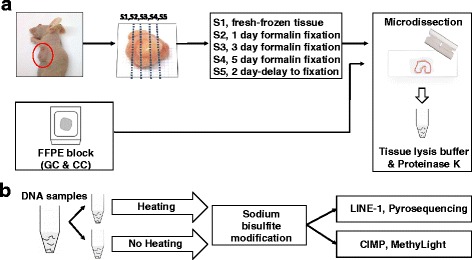
**a** DNA preparation of mouse xenograft tissue samples (*n* = 10) and human archival tissue samples (gastric cancer (GC), *n* = 476; colorectal cancer (CC), *n* = 497). Each xenograft tumor tissue was cut into five slices. Each slice was treated using five different formalin fixation protocol. **b** Both heat-treated and heat-untreated DNA samples were subjected to bisulfite conversion and subsequent pyrosequencing methylation assay or MethyLight assay

Each xenograft cancer tissue (*n* = 10) was cut into five slices, which were treated with five different durations of formalin fixation (no fixation, 1-day fixation, 3-day fixation, 5-day fixation, or a 2-day delay prior to 1-day fixation). Ten-micrometer sections cut from the FFPE tissue blocks or from the FF sections were stained with hematoxylin and eosin and examined under a microscope. Tumor areas with the highest tumor cell density were scraped into microtubes containing lysis buffer solution. The lysed tissue solution was divided into two halves, one of which was treated with heating (95 °C for 30 min) and the second with no heating step. The overall experimental design was indicated in Fig. 1.

### Effect of formalin fixation in the assessment of LINE-1 methylation

**Fig. 2:**
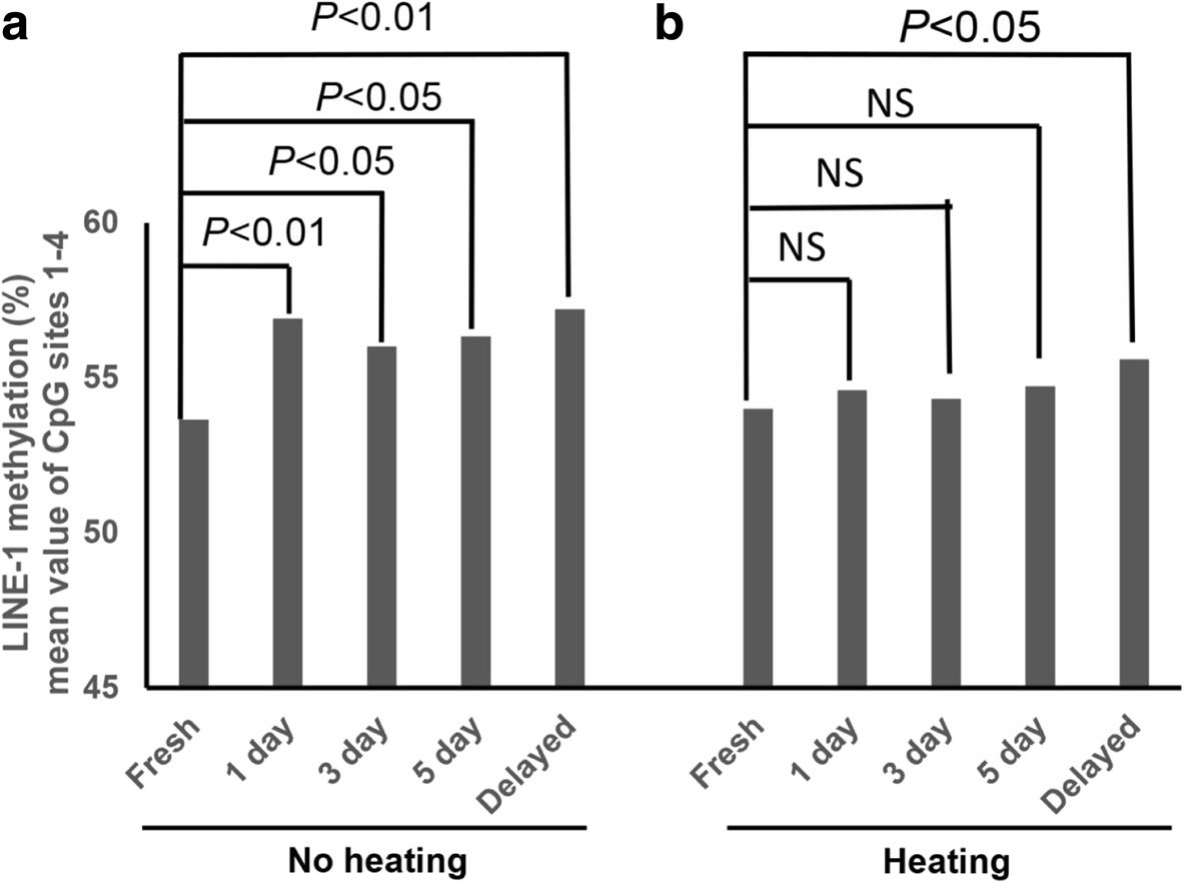
Mean methylation levels of the four LINE-1 CpG sites for xenograft tissue samples (*n* = 10) with five different durations of formalin fixation. DNA samples were heat-untreated (**a**) and heat-untreated (**b**). Both the paired Student’s *t* test and the paired Wilcoxon signed-rank test were performed to compare the mean methylation values of the four CpG sites between paired fresh-frozen and formalin-fixed, paraffin-embedded tissue samples. *P* values in the bar graph represent the values of both the parametric and the non-parametric tests

When the LINE-1 methylation level was compared between paired fresh and formalin-fixed tissues from xenograft tissue slices, FFPE tissue samples showed increased levels of LINE-1 methylation compared with paired FF tissue samples. Regardless of the duration of formalin fixation, FFPE tissue samples exhibited significantly higher values of methylation than those of FF tissue samples (56.6 vs. 53.7%) (Fig. 2). No difference was noted in the values of LINE-1 methylation level among FFPE tissue samples across the various fixation parameters.

### Heating decreases the formalin-associated elevation of LINE-1 methylation levels

The discrepancy of LINE-1 methylation values between paired FF and FFPE tissue samples was found to decrease with the application of a heating step (95 °C, 30 min) during the FFPE DNA extraction process. With the addition of a heating step during DNA preparation, the differences in the measured levels of LINE-1 methylation between paired FF and FFPE tissue samples became insignificant (Fig. 2). However, FFPE tissue samples with delayed fixation showed significantly increased measured values of the LINE-1 methylation level despite the application of a heating step. The correlation of the measured values became stronger between paired FF and FFPE tissue samples with the application of a heating step. Although we tested another condition of heating, heating at 95 °C for 1 h, we did not find any difference in LINE-1 methylation levels between samples treated with heating at 95 °C for 30 min and those with heating at 95 °C for 1 h (Additional file 1: Figure S1).

### Effect of heating on LINE-1 methylation analysis of archival tissue samples and survival analysis

**Fig. 3:**
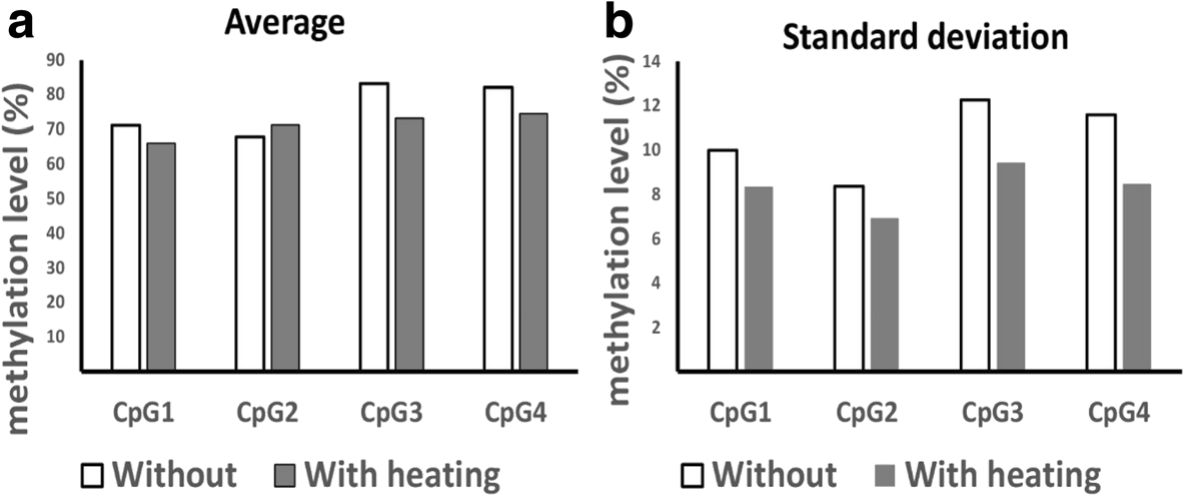
Comparison of the methylation levels (**a**) and the standard deviations (**b**) in the four LINE-1 CpG sites of gastric cancer tissue DNA samples (*n* = 476) with and without heat treatment

**Fig. 4:**
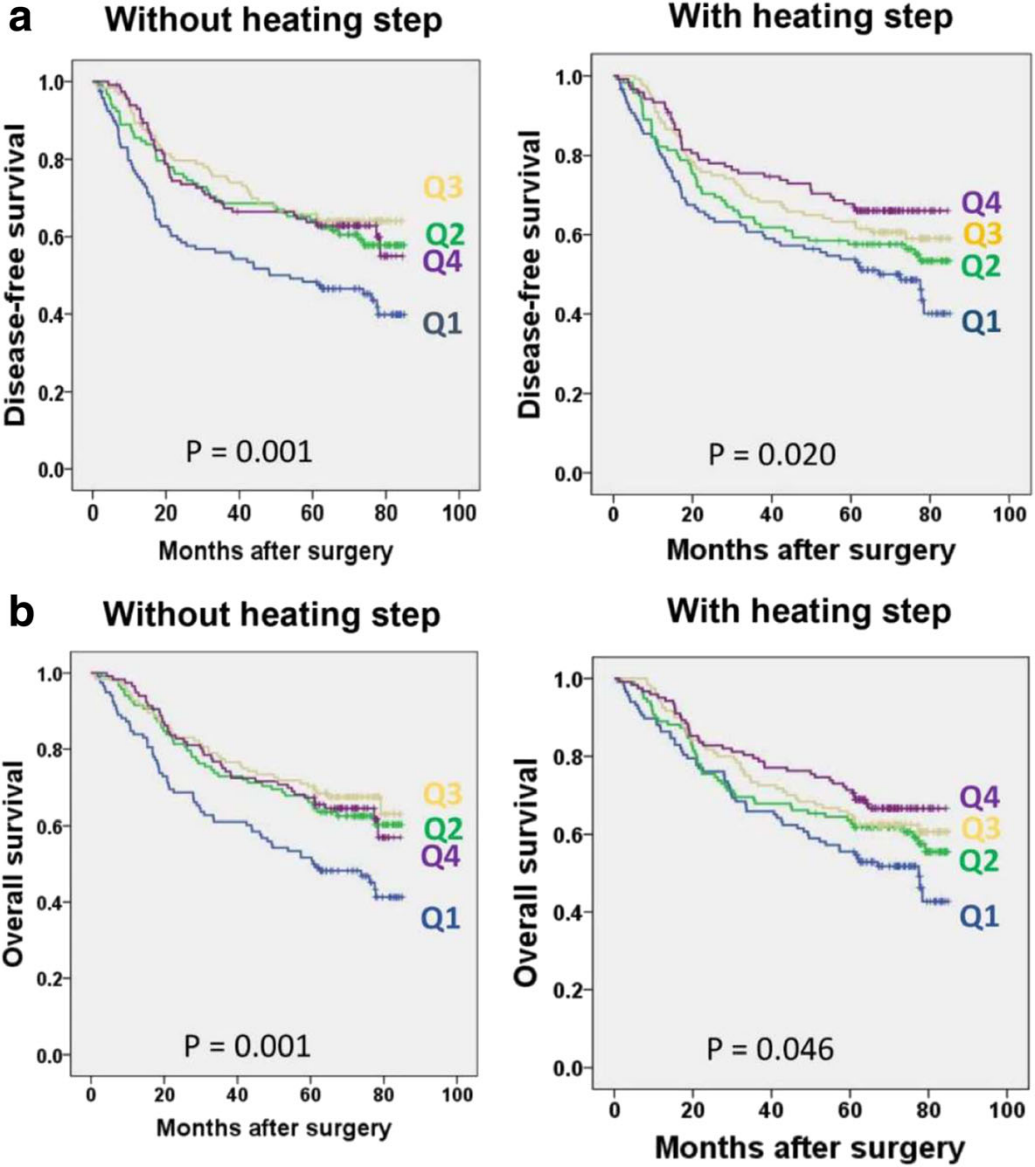
Gastric cancer patients (*n* = 476) were divided into four groups according to their tumoral LINE-1 methylation levels and its association with dissected-free survival (**a**) and overall survival (**b**) was observed. Q1, Q2, Q3, and Q4 are in the order of increasing LINE-1 methylation levels

Methylation assays for four LINE-1 CpG sites were performed on both heat-treated and untreated DNAs from 476 cases of advanced gastric cancer. When the methylation levels of four individual LINE-1 CpG sites were compared between advanced gastric carcinoma DNA samples with and without heat, all but CpG site 2 showed decreased values of methylation with concomitant decrease in the standard deviation of the measured value in all four CpG sites (Fig. 3). When advanced gastric cancer cases were split into four groups according to their tumor LINE-1 methylation levels, different survival curves were observed for each group after the application of a heating step during DNA extraction compared to unheated extractions. In the survival analysis of heat-treated DNA samples, a lower LINE-1 methylation level correlated with decreased progression-free survival (PFS) and overall survival (OS) (Fig. 4).

### Effect of heating on the methylation analysis of single genes in archival tissue samples

**Fig. 5:**
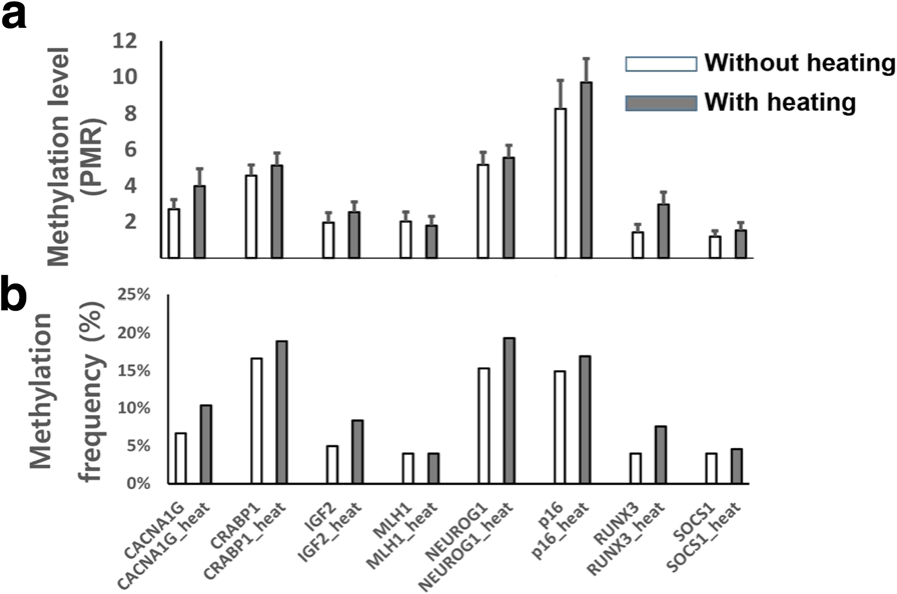
Comparison of the methylation levels (**a**) and frequencies (**b**) in the eight individual CpG island methylator phenotype panel markers in colorectal cancer tissue (*n* = 497) DNA samples with and without heat treatment

To identify whether the application of heat during DNA extraction might affect the results of single gene methylation analysis, we compared the methylation levels and frequencies of eight CpG island methylator phenotype (CIMP) panel markers between DNA samples with and without heat treatment. The samples from 497 colorectal cancer cases were split into two, and CIMP analysis was performed on paired heat-treated and untreated DNA samples using the MethyLight assay. Of the eight markers, all but MLH1 showed increased methylation frequencies and levels in the DNA samples subjected to heat treatment during extraction compared with DNA samples without heat treatment (Fig. 5). The application of heat during DNA extraction thus resulted in enhanced detection of CIMP-high CRC (Additional file 2: Figure S2).

## Discussion

FFPE tissue is an invaluable resource for oncologic epigenetic studies because large collections of FFPE tissue samples with clinical annotations are managed in hospitals. Histologic examination and delineation of areas with the highest tumor cell content is important for the quantitative methylation analysis of cancers to prevent dilution of the neoplastic cell signal by contributions from non-neoplastic cells in the cancer tissue samples. FFPE tissue samples are more suitable for manual microdissection than FF tissue samples. Minimal amounts of tissue, even a 10-μm-thick section from an endoscopic biopsy specimen, are required for the PCR-based quantitative methylation analysis of single genes or repetitive DNA elements. Tissue samples dissected from the mounted tissue slices are subjected to incubation in lysis buffer containing proteinase K at 55 °C for 2 days. Then, the lysed tissue solutions are subjected to bisulfite conversion using commercially available kits, including the EpiTect or EZ DNA methylation kit, which generate bisulfite-modified DNA samples. In our study, the addition of a heating step, 95 °C for 30 min, between the 2-day incubation step and the bisulfite conversion procedure was found to decrease the discrepancy between the measured values of LINE-1 methylation of paired fresh and fixed tissue samples.

In the present study, formalin fixation was found to increase the measured value of LINE-1 methylation levels compared to the paired FF xenograft tissue samples. But, the measured value of the LINE-1 methylation levels was not different among FFPE xenograft tissue samples of varying fixation lengths from 1 to 5 days. These findings suggest that formation fixation, rather than duration of fixation, plays an important role in the causation of deviation in the measured value of LINE-1 methylation level. However, because we did not examine the effect of fixation duration >7 days or <1 day on the performance of pyrosequencing-based LINE-1 methylation assay, we could not insist that the duration of fixation does not affect the pyrosequencing assay of LINE-1 methylation. However, to the best of our knowledge, there has been no study which investigated how duration of formalin fixation affects bisulfite conversion of genomic DNA and its performance in downstream methylation analysis. Formaldehyde reacts with amino groups in nucleobases, leading to the formation of methylene bridges between complementary strands of DNA. Because the generation of methylene crosslinks is a time-dependent process [17], longer fixation time is expected to form more inter-strand crosslinks. However, we did not find any difference in the measured value of LINE-1 methylation among fixation time from 1 to 5 days.

As a surrogate marker for genomic DNA methylation content, methylation of LINE-1 has been measured by various assays, including the combined bisulfite restriction assay [18], the MethyLight assay [2], pyrosequencing, and absolute quantitative analysis of methylated alleles [19], which target CpG sites located in the 5′ untranslated region of LINE-1. However, 85% of LINE-1 elements are truncated in their 5′ sequences [20], and thus, PCR-based assays only assess 15% of the LINE-1 elements in the human genome. Weisenberger et al. demonstrated that the MethyLight assay-based measurement of LINE-1 methylation level correlates with genomic DNA methylation content as measured by high-performance liquid chromatography (HPLC). However, before the study of Lisanti et al. [21], there had been no study that directly analyzed the correlation between the pyrosequencing-based measurement of LINE-1 methylation and genomic DNA methylation content. Lisanti et al. showed a strong correlation between the methylation levels in LINE-1 measured via a pyrosequencing assay versus a high-performance liquid chromatography.

Studies have shown that hypomethylation of LINE-1 in tumors is closely associated with poor patient prognosis for many types of human cancers, including gastric carcinoma [6, 22], colorectal carcinoma [9], esophageal squamous cell carcinoma [23], and lung adenocarcinomas [24, 25]. In particular, the association between LINE-1 hypomethylation in gastric carcinoma and poor prognosis has been reported by three studies [6, 11, 22]. However, all three of these studies, including Shigaki et al. [6], used FFPE tissue samples. In the Shigaki study, DNA was extracted from FFPE tissue samples using the QIAamp DNA FFPE tissue kit, which includes a heating step for 60 min at 90 °C in its protocol. However, our previous studies did not use commercial DNA purification kits, and the extraction protocol did not include any prolonged heating step. In the present study, we analyzed the effect of heat during DNA extraction from FFPE tissue samples by comparing the survival curves of four groups for both heat-treated DNAs and untreated DNA. With heat-treated DNA samples, PFS and OS decreased as tumoral LINE-1 methylation decreased. This trend was not observed with unheated DNA samples. Regardless of whether the heating step was included during DNA extraction, tumoral LINE-1 hypomethylation was found to be an independent parameter for identifying gastric cancer patients with a poor prognosis.

When we examined the methylation frequencies and levels of eight CIMP panel markers between DNA samples with and without heat treatment, all of the genes except for MLH1 showed increased methylation frequencies and levels in the heat-treated DNA samples relative to the unheated DNA samples. Consequently, heat treatment enabled identification of more cases of CIMP-high CRCs. At present, it is unclear why assessment of methylation at the MLH1 CpG island locus was not affected by heat treatment. Interestingly, the increased methylation level of individual genes in association with heat treatment is in contrast to the decreased methylation levels of LINE-1 under the same conditions. The question arises why heat treatment led to a decrease in methylation levels in repetitive DNA elements but an increase in individual genes. The reason of this apparent discrepancy is unclear, but it might be attributable to the difference in the methylation assays employed: LINE-1 methylation was analyzed using a pyrosequencing assay, which measures the mean methylation level of all the DNA alleles at individual CpG sites [2], whereas the methylation levels of individual genes were assessed by the MethyLight assay, which evaluates the relative amount of specific DNA alleles with concurrent methylation of serial CpG sites [26]. When the region of interest is incompletely converted and thus contains non-converted CpG and non-CpG cytosines, the MethyLight probe cannot anneal to the incompletely converted region of interest and does not generate a fluorescent signal. Thus, on the condition that all CpG sites located in the region of interest are methylated, complete conversion leads to an increase in the measured value of methylation levels compared to an incomplete conversion. In contrast with the MethyLight assay, enzymatic cascade of the pyrosequencing reaction cannot discriminate incompletely the converted template sequence (region of interest) and generates luciferase light signals not only from methylated CpG cytosines but also from non-converted unmethylated CpG cytosines, which causes an increase in the measured value of methylation levels compared to complete conversion.

## Conclusions

We have demonstrated that formalin fixation can result in elevated values of the LINE-1 methylation level, irrespective of the duration of fixation, but prolonged heating of the DNA solution prior to bisulfite conversion helps decrease the discrepancy between paired FF and FFPE tissue samples. However, heating did not offset the discrepancy in FFPE tissue samples prepared using delayed fixation. Our results indicate that prolonged heating of DNA samples obtained from FFPE tissues is necessary for proper evaluation of DNA methylation levels, regardless whether single genes or repetitive DNA elements are assayed.

## Methods

### Cell culture

The human gastric cancer cell lines, MKN-45 and SNU-638, and colorectal cancer cell lines, SW620, SNU-C5, and LoVo, were obtained from the Korean Cell Line Bank (Seoul, Korea) and were cultured in a 37 °C incubator with 5% CO_2_. Cells were grown in RPMI 1640 with 10% heat-inactivated fetal bovine serum, 100 U/mL penicillin and 100 μg/mL streptomycin. Culture medium was replaced approximately every 48 h.

### In vivo xenograft experiment

A total of 10 6-week-old normal BALB/c-nu mice were used for the tumor xenograft experiment. To generate two tumor masses per mouse, 2 × 10^6^ cells in phosphate-buffered saline were bilaterally injected subcutaneously into the flanks of the mice. The mice were maintained in a pathogen-free barrier facility and fed a standard diet. All mice were euthanized 8 to 10 weeks after subcutaneous injection. The xenograft experimental plan and protocol were approved by the Biomedical Research Institute of Seoul National University Hospital (15-0111-C1A0).

### DNA extraction from xenograft tissue samples

FFPE tissue mouse xenograft tissue slides were made according to one of five formalin-fixation conditions: no fixation (fresh frozen), fixation for 1, 3, or 5 days; or delayed fixation (room temperature for 2 days prior to fixation for 1 day). Ten-micrometer-thick sections were cut from the FFPE tissue blocks or from the FF tissue blocks. Deparaffinization was accomplished by first heating the glass slides mounted with the paraffin section to no more than 60 °C. The paraffin was dissolved in xylene. Deparaffined and rehydrated sections of the FFPE tissue blocks or sections of the FF tissue blocks were stained with hematoxylin and eosin. The FFPE or FF tissue slides were examined under a microscope, and the areas with the highest tumor cell density were selectively dissected using a knife blade. The scraped tissues were collected in microcentrifuge tubes containing 50 μL of tissue lysis buffer (0.5% Tween 20 (Sigma, St Louis, MO, USA), 100 mM Tris HCl buffer (pH 7.6), 1 mM EDTA, and 20 μg of proteinase K (Sigma)). After incubation at 55 °C for 2 days to ensure complete lysis, the microcentrifuge tubes were centrifuged at 10,000×*g* for 1 min to remove insoluble debris. The supernatant was transferred to a newly labeled microcentrifuge tube. DNA samples were prepared from the human FFPE tissue blocks using the same protocol for the xenograft FFPE tissue blocks.

### Patient specimens

We retrospectively analyzed the clinicopathologic data of 476 patients who underwent surgery and extended lymph node dissection (D2) for advanced gastric cancer in the Seoul National University Hospital, Seoul, Korea, from January 2007 to December 2008. Patients who had a history of other primary malignancies within 5 years or were treated with neoadjuvant chemotherapy were excluded. The following pathological parameters were evaluated by gross and microscopic examination: tumor location, tumor differentiation, histological type, lymphatic invasion, perineural invasion, venous invasion, and TNM stage (American Joint Committee on Cancer, 7th edition). A total of 497 colorectal cancer patients who received curative surgery and adjuvant chemotherapy in the Seoul National University Hospital between June 2005 and November 2011 were included. Because each FFPE tissue block was made soon after the surgery, the tissue blocks of gastric cancer and colorectal cancer ranged in age from 6 to 7 and 4 to 10 years, respectively, at the time of DNA extraction. Microscopically, tumor areas with high tumor density and representative histology were marked for each case, were manually dissected, and were collected into microcentrifuge tubes containing tissue lysis buffer and proteinase K. The tissue solution was kept at 55 °C for 2 days.

The study protocol was reviewed and approved by the institutional review board of Seoul National University Hospital (1312-051-542) and was performed in accordance with the recommendations of the Declaration of Helsinki (2013) for biomedical research involving human subjects. Patient records/information were anonymized and de-identified prior to analysis.

### Bisulfite conversion and Alu-based MethyLight control reaction

We used 20 μL of the supernatant for the bisulfite modification which was performed using the EZ DNA methylation kit according to the manufacturer’s protocol (Zymo Research, Irvine, CA, USA). In order to measure input DNA (bisulfite-modified DNA), we performed Alu-based MethyLight control reaction which is a CpG-independent, bisulfite-specific control reaction [2]. We determined the threshold cycle (C(t) value) of this reaction in which the Alu reaction fluorescence was detected. To keep the C(t) value of bisulfite-modified DNA samples in the range from 18 to 20, we added distilled water to dilute bisulfite-modified DNA samples with C(t) values lower than 18. MethyLight PCR was performed in a 25-μL reaction volume with 200 μM dNTPs, 0.3 μM forward and reverse PCR primers, 0.1 μM probe, 3.5 mM MgCl_2_, 0.01% Tween 20, 0.05% gelatin, and 0.2 units of Taq polymerase on a 96-well plate (BioRad) using the following PCR program: 95 °C for 10 min, then 50 cycles of 95 °C for 15 s followed by 60 °C for 1 min.

### Pyrosequencing methylation assay

The converted DNA samples were PCR-amplified with oligonucleotide primers that were designed against a consensus LINE-1 sequence by the Issa group for pyrosequencing [13]; the forward primer was 5′-TTTTGAGTTAGGTGTGGGATATA, and the reverse biotinylated primer was 5′-biotin-AAAATCAAAAAATTCCCTTTC. The PCR reaction was carried out in a 25-μL final volume comprised of 2 μL of bisulfite-treated DNA (input DNA was approximately 33 ng), 2.5 μL of CoralLoad PCR Buffer, 1.5 μL of 25 mM MgCl_2_, 1 μL of the forward and biotinylated reverse primers (0.4 μM final concentration), and 0.75 U of HotStarTaq Plus DNA polymerase (Qiagen, Valencia, CA, USA). The PCR cycling conditions were as follows: initial denaturing at 95 °C for 10 min, 50 cycles of 94 °C for 30 s, 57 °C for 40 s, and 72 °C for 40 s followed by a final extension at 72 °C for 5 min. The PCR products were added to the binding buffer (Qiagen) and the Streptavidin Sepharose High Performance beads (GE Healthcare Bio-Sciences Corp., Uppsala, Sweden). The biotinylated DNA-bound beads were collected and retained using the PyroMark Vacuum Prep WorkStation (Qiagen). The purified single-stranded PCR product was added to the annealing buffer (Qiagen) with 0.3 μM of sequencing primer (5′-AGTTAGGTGTGGGATATAGT), and the pyrosequencing reaction was performed using the PyroMark Q24 platform (Qiagen). The level of methylation at each of the four analyzed CpG sites (GenBank accession number X58075 sites 1–4: nucleotide positions 328, 321, 318, and 306) was determined by the percentage of methylated cytosines. The pyrosequencing assay was repeated in triplicate, and the median value of the three replicates was reported as the representative value of LINE-1 methylation.

### Measurement of single gene methylation level

For evaluation of the DNA methylation status in individual genes, the MethyLight assay was performed as previously described [27]. The converted DNA samples were analyzed for methylation status in eight individual genes (*CACNA1G*, *CDKN2A* (*p16*), *CRABP1*, *IGF2*, *MLH1*, *NEUROG1*, *RUNX3*, and *SOCS1*). A complete list of MethyLight reaction probes and primers has been previously reported [28]. The MethyLight assay was repeated in triplicate, and the median methylation level (determined by the percentage of methylated reference (PMR)) was obtained.

### Statistical analysis

All statistical analyses were performed using SPSS for Windows (version 21.0) (International Business Machines Corp., Armonk, NY, USA). Two-sided *P* values <0.05 were considered significant. The clinical database for gastric cancer patients was updated in January 2014. Progression-free survival (PFS) was calculated from the date of resection of advanced gastric cancer to the first date of documented recurrence or the date of death from any cause. Overall survival (OS) was measured from the date of resection to the date of death or the date of the last clinical follow-up before January 2014. Kaplan-Meier survival analysis was performed to compare OS and PFS using the log-rank test. Because the data on the level of LINE-1 methylation in CpG sites 1 to 4 did not follow the normal distribution, mean values across two or more groups were compared using both parametric and non-parametric tests. The Mann-Whitney *U* test and ANOVA test were used for the comparison of mean values across two groups, while the Kruskal-Wallis test and Student’s *t* test were used for the comparison across three or more groups. Pearson’s correlation test was used to assess the correlation between the LINE-1 methylation levels in paired FF and FFPE xenograft tissue samples. The Wilcoxon signed-rank test and paired Student’s *t* test were used to analyze the paired differences.

## Additional files

Additional file 1: Figure S1. Methylation levels of the four LINE-1 CpG sites for four xenograft DNA samples (MKN-45, SNU-638, SW620, and LoVo xenograft tumors) with heating at 95 °C for 30 or 60 min. Both the paired Student’s *t* test and the paired Wilcoxon signed-rank test were performed to compare the mean methylation values of the four CpG sites between paired fresh-frozen and formalin-fixed, paraffin-embedded tissue samples. *P* values in the bar graph represent the values of both the parametric and the non-parametric tests. (PDF 244 kb)
Additional file 2: Figure S2. (A) Application of a heating step during DNA extraction increased the mean number of methylated CIMP panel markers from 0.8 to 0.9. Cases with no methylation of CIMP panel markers decreased from 66.9 to 61.2%. (B) Application of a heating step allowed identification of 1.8% more CIMP-high colorectal cancer cases. (PDF 238 kb)
