# Supplementary figures and images for: Improved results of LINE-1 methylation analysis in formalin-fixed, paraffin-embedded tissues with the application of a heating step during the DNA extraction process
Source: Clin Epigenetics. 2017 Jan 13;9:1. doi: 10.1186/s13148-016-0308-0 (PMC5270344; doi:10.1186/s13148-016-0308-0)

Supplementary Fig. 1

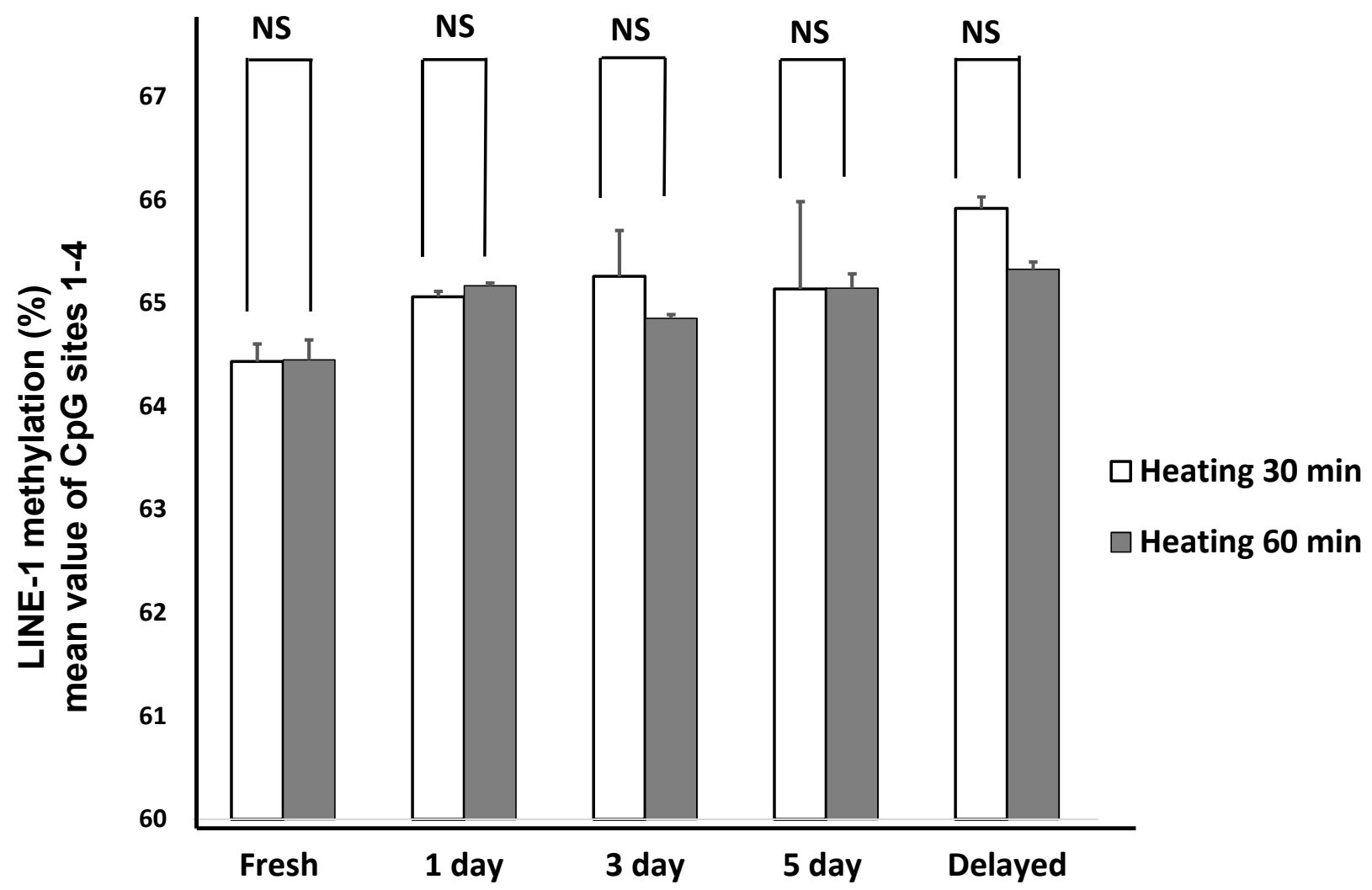

Supplement: Additional file 1: Figure S1. — Methylation levels of the four LINE-1 CpG sites for four xenograft DNA samples (MKN-45, SNU-638, SW620, and LoVo xenograft tumors) with heating at 95 °C for 30 or 60 min. Both the paired Student’s t test and the paired Wilcoxon signed-rank test were performed to compare the mean methylation values of the four CpG sites between paired fresh-frozen and formalin-fixed, paraffin-embedded tissue samples. P values in the bar graph represent the values of both the parametric and the non-parametric tests. (PDF 244 kb) [file 13148_2016_308_MOESM1_ESM.pdf]

# Supplementary Fig. 2

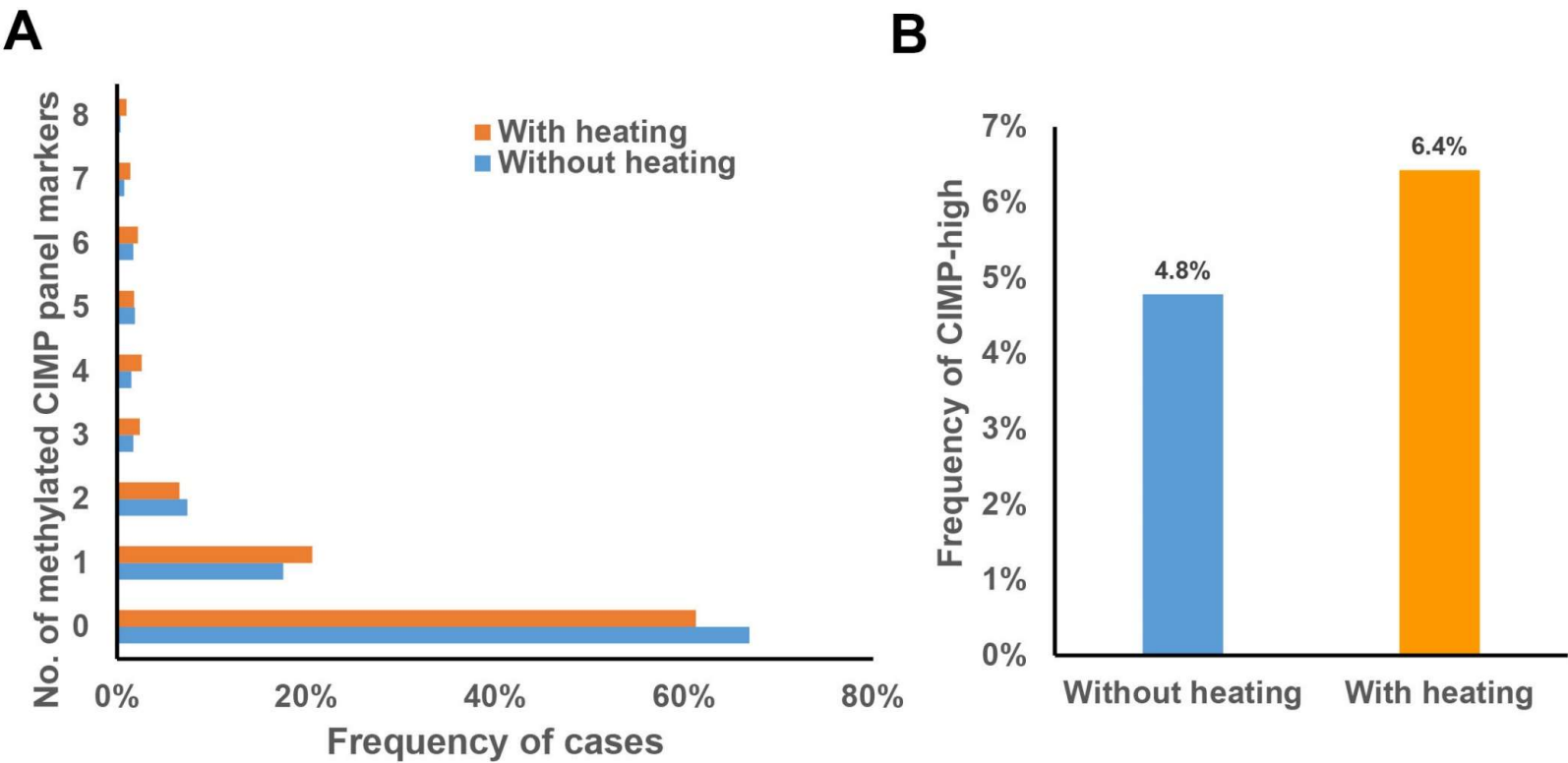

Supplement: Additional file 2: Figure S2. — (A) Application of a heating step during DNA extraction increased the mean number of methylated CIMP panel markers from 0.8 to 0.9. Cases with no methylation of CIMP panel markers decreased from 66.9 to 61.2%. (B) Application of a heating step allowed identification of 1.8% more CIMP-high colorectal cancer cases. (PDF 238 kb) [file 13148_2016_308_MOESM2_ESM.pdf]
